# Mitophagy-associated programmed neuronal death and neuroinflammation

**DOI:** 10.3389/fimmu.2024.1460286

**Published:** 2024-10-02

**Authors:** Yanlin Zhu, Jianning Zhang, Quanjun Deng, Xin Chen

**Affiliations:** ^1^ Department of Neurosurgery, Tianjin Medical University General Hospital, Tianjin, China; ^2^ Tianjin Neurological Institute, Key Laboratory of Post-Trauma Neuro-Repair and Regeneration in Central Nervous System, Ministry of Education, Tianjin Key Laboratory of Injuries, Variations and Regeneration of Nervous System, Tianjin, China; ^3^ Tianjin Key Laboratory of Injuries, Variations and Regeneration of Nervous System, Tianjin, China

**Keywords:** mitophagy, apoptosis, necroptosis, pyroptosis, ferroptosis, neuroinflammation

## Abstract

Mitochondria are crucial organelles that play a central role in cellular metabolism and programmed cell death in eukaryotic cells. Mitochondrial autophagy (mitophagy) is a selective process where damaged mitochondria are encapsulated and degraded through autophagic mechanisms, ensuring the maintenance of both mitochondrial and cellular homeostasis. Excessive programmed cell death in neurons can result in functional impairments following cerebral ischemia and trauma, as well as in chronic neurodegenerative diseases, leading to irreversible declines in motor and cognitive functions. Neuroinflammation, an inflammatory response of the central nervous system to factors disrupting homeostasis, is a common feature across various neurological events, including ischemic, infectious, traumatic, and neurodegenerative conditions. Emerging research suggests that regulating autophagy may offer a promising therapeutic avenue for treating certain neurological diseases. Furthermore, existing literature indicates that various small molecule autophagy regulators have been tested in animal models and are linked to neurological disease outcomes. This review explores the role of mitophagy in programmed neuronal death and its connection to neuroinflammation.

## Introduction

**Figure 1:**
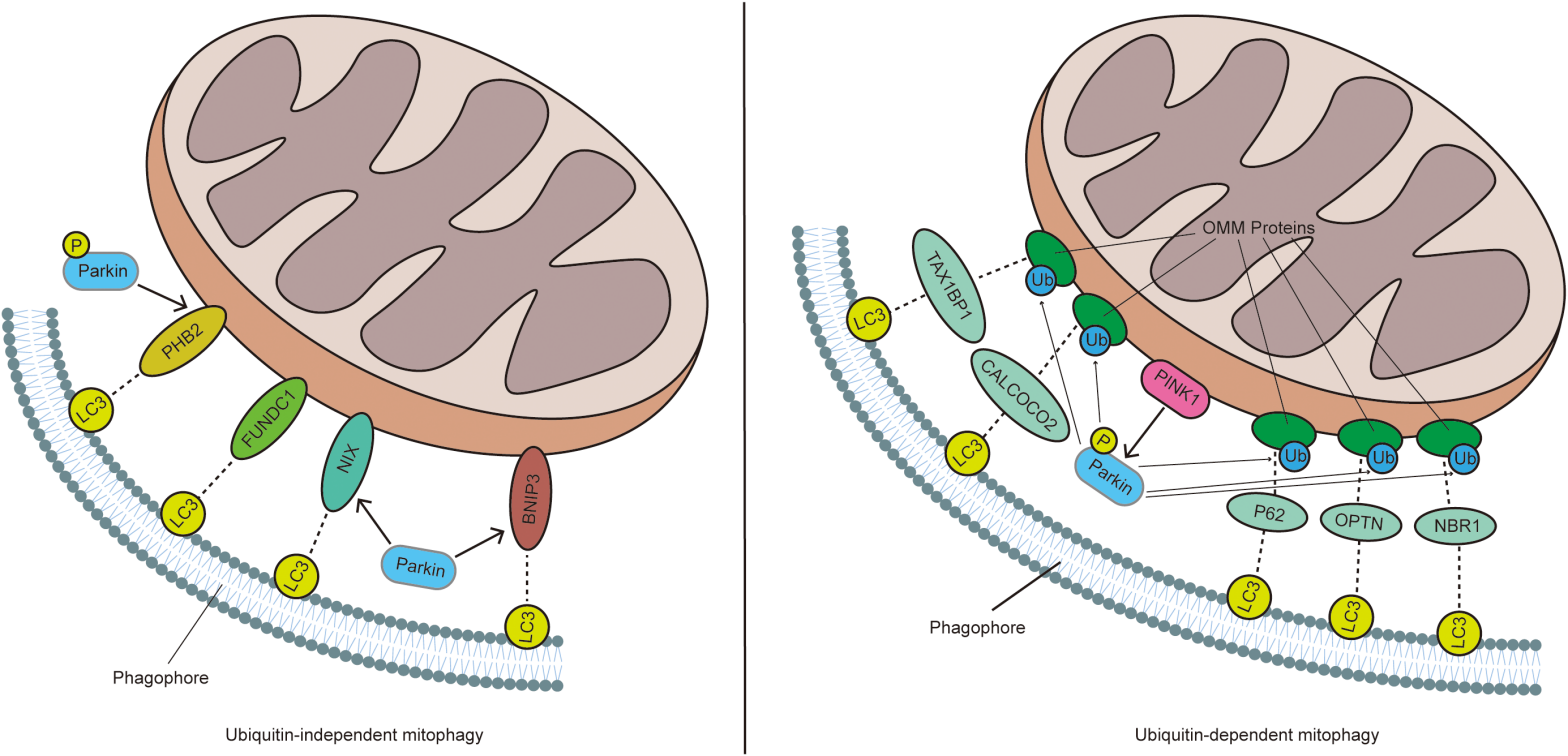
In ubiquitin-dependent mitophagy, a decrease in MMP leads to the accumulation of PINK1 on the OMM, which promotes the recruitment and phosphorylation of Parkin. Activated Parkin ubiquitinates mitochondrial proteins, which are then recognized by receptors such as P62, NBR1, OPTN, TAX1BP1, and CALCOCO2. These receptors interact with LC3 through their LIR domain, thereby inducing mitophagy. In contrast, in ubiquitin-independent mitophagy, mitophagy receptors like BNIP3, NIX, PHB2, and FUNDC1 are directly anchored to the OMM and mediate mitophagy by directly interacting with LC3, without the need for ubiquitination.

Mitochondria are essential organelles responsible for cellular metabolism and function, generating the majority of the cell’s energy while also regulating cell growth and apoptosis. Damage to these structures can lead to cellular defects and is associated with various diseases (1). Often referred to as the cell’s power source, mitochondria produce most of the ATP required by cells, making them indispensable for eukaryotic life. However, during the functioning of mitochondria, a significant amount of reactive oxygen species (ROS) are generated, which can damage mitochondrial DNA (mtDNA) (2). When mitochondria are damaged, they can release high levels of Ca^2+^ and cytochrome c into the cytoplasm, triggering cell apoptosis. Mitophagy, a vital mechanism for maintaining mitochondrial quality, ensures the clearance of damaged mitochondria, which is crucial for preserving cellular and metabolic homeostasis and, ultimately, cell survival (3). Depending on the signals that target damaged or excess mitochondria for degradation, mitophagy can be categorized into four main types: ubiquitin-dependent mitophagy, ubiquitin-independent or receptor-mediated mitophagy, lipid-based mitophagy, and micromitophagy. Of these, ubiquitin-dependent and ubiquitin-independent mitophagy are the most prevalent (4) (**Figure 1**).

### Ubiquitin−dependent mitophagy

As early as 2006, it was discovered that phosphatase and tensin homolog (PTEN)-induced kinase 1 (PINK1) and Parkin operate within the same pathway, playing a crucial role in maintaining mitochondrial function (5, 6). Under normal conditions, PINK1 is imported into the mitochondria via the outer membrane translocase (TOM) complex and the inner membrane translocase (TIM23) complex, facilitated by its amino-terminal mitochondrial targeting sequence. Once inside, PINK1 enters the inner mitochondrial membrane (IMM) complex (7). In the mitochondrial matrix, the mitochondrial processing peptidase (MPP) cleaves the N-terminal mitochondrial targeting sequence of PINK1, while presenilin-associated rhomboid-like protein (PARL) further cleaves the M-segment of PINK1 (8, 9). The remaining PINK1, now with an unstable N-terminus, is released into the cytoplasm, where it is degraded by the proteasome pathway involving cytoplasmic ubiquitin ligase E3 components N-recognin 1 (UBR1), UBR2, and UBR4. During this process, Parkin E3 ubiquitin ligase, typically in an inactive state, remains in its natural self-inhibitory conformation within the cytoplasm (10). When mitochondria are damaged, a decrease in mitochondrial membrane potential (MMP) triggers the self-phosphorylation of PINK1 at Ser228 and Ser402 residues. This self-phosphorylation event recruits the E3 ubiquitin ligase Parkin to the mitochondria, altering its spatial conformation and converting it into an active E3 ubiquitin ligase (11). Activated Parkin ubiquitinates several mitochondrial proteins, including mitofusin 1 (Mfn1), mitofusin 2 (Mfn2), voltage-dependent anion-selective channel protein (VDAC), and dynamin-1-like protein (DRP-1) (12). These ubiquitinated proteins on the outer mitochondrial membrane (OMM) then recruit various autophagic receptors, such as sequestosome 1 (P62/SQSTM1) (13), neighbor of BRCA1 gene 1 (NBR1) (14), calcium binding and coiled-coil domain 2 (CALCOCO2/NDP52) (15), tax1 binding protein 1 (TAX1BP1) (16), and optineurin (OPTN) (17). Microtubule-associated protein light chain 3 (LC3) recognizes these receptors through the LC3-interacting region (LIR) motif and subsequently transports the dysfunctional mitochondria to autophagosomes. These autophagosomes then fuse with lysosomes, allowing the degraded mitochondria to be processed and cleared from the cell (18).

Additionally, multiple signaling pathways influence mitophagy by regulating the expression of PINK1/Parkin. Autophagy and Beclin 1 regulator 1 (AMBRA1) is essential for the effective activation of PINK1-PRKN (Parkin) signaling during mitochondrial depolarization. In the absence of AMBRA1, the autophagic response to mitochondrial damage is impaired (19). The PINK1/Parkin pathway acts as a signal transduction mechanism, labeling damaged mitochondria with ubiquitin chains and recruiting mitophagy receptors. These receptors guide the autophagosome membrane to envelop the ubiquitinated mitochondria, which are then eliminated through autophagosome-lysosome pathways (20). Several factors have been found to modulate this pathway. For instance, LncRNA H19 can inhibit the PINK1/Parkin signaling pathway, thereby alleviating cardiac defects associated with obesity (21). In diabetic retinopathy, high glucose levels promote apoptosis of retinal pigment epithelial (RPE) cells and inhibit mitophagy by downregulating ROS-mediated PINK1/Parkin signaling (22). Stomatin-like protein 2 (STOML2) interacts with and stabilizes PINK1, amplifying PINK1/Parkin-mediated mitophagy, which in turn promotes the growth and metastasis of hepatocellular carcinoma (HCC) (23). Follicle-stimulating hormone (FSH) protects granulosa cells (GCs) from hypoxic damage by activating PINK1/Parkin-mediated mitophagy (24). Conversely, inhibiting extracellular signal-regulated protein kinases (ERK)1/2 signaling can block docosahexaenoic acid (DHA)-mediated mitophagy (25). Melatonin has also been shown to mitigate the aging of renal tubular epithelial cells induced by glyphosate and hard water by upregulating PINK1/Parkin-dependent mitophagy (26).

### Ubiquitin-independent mitophagy (receptor-dependent)

In contrast to PINK1/Parkin-mediated ubiquitination of mitophagy, several proteins located on OMM contain LC3-interacting region (LIR) motifs and act as autophagy receptors. These receptors are typically expressed on the OMM surface and can directly bind to LC3 without the need for ubiquitination, thereby initiating mitophagy (27). LC3-I is typically converted to LC3-II during mitophagy, and the LC3-II/I ratio is commonly used to reflect changes in mitophagy levels. A higher LC3-II/I ratio indicates a higher level of mitophagy (28). Key receptors that facilitate this process include Bcl2-interacting protein 3 (BNIP3), BNIP3-Like (BNIP3L)/NIP3-like protein X (NIX), prohibitin 2 (PHB2), and FUN14 domain containing 1 (FUNDC1) (29).

BNIP3, encoded by the Bnip3 gene located on human chromosome 10q26.3 and initially referred to as “NIP3”, is believed to be part of the Bcl-2 family, which regulates cell death. It contains an atypical BH3 domain, characteristic of certain Bcl-2 family proteins (30). BNIP3 is a pro-apoptotic protein that functions through the mitochondrial pathway and can induce cell death in various cell lines (31, 32). It serves as an integral node for regulating mitophagy via endosomes and proteasomes (33). In patients with EB virus-positive diffuse large B-cell lymphoma, silencing of CD30 leads to mitochondrial dysfunction and inhibits mitophagy. This inhibition, in turn, reduces the expression of BNIP3, resulting in the accumulation of damaged mitochondria and promoting tumor cell apoptosis (34). In rheumatoid arthritis (RA) patients, the activation of fibroblast-like synoviocytes (FLS) under hypoxic conditions involves BNIP3-mediated mitophagy. Additionally, autophagy can eliminate ROS and inhibit the HIF-1α/NLRP3 pathway, thereby reducing hypoxia-induced FLS pyroptosis (35). DHA regulates mitophagy through the PPARγ-LC3-BNIP3 pathway, inducing apoptosis in cells, reducing adipocyte numbers, and inhibiting lipid accumulation (36). Furthermore, 6-Gingerol exerts a protective effect against placental injury in preeclampsia by reducing oxidative stress and inhibiting excessive mitophagy caused by mitochondrial dysfunction (37).

BNIP3L/NIX is an OMM protein that belongs to the Bcl2 family of BH3-only proteins. BNIP3L acts as a mitophagy receptor by binding to LC3 through its N-terminal LIR motif (38). This interaction with ATG8 family proteins in LC3 initiates mitophagy (39). In a study involving mice, pretreatment with a NIX enhancer before corticosterone exposure increased mitophagy and synaptic density in the hippocampus, leading to improved performance in spatial memory tasks (40). Direct phosphorylation of NIX by PRKA/PKA can reverse NIX-induced mitophagy, causing BNIP3L to translocate from the mitochondria to the cytoplasm (41). FBXL4 interacts with BNIP3 and NIX, controlling their stability; a deficiency in FBXL4 ubiquitin ligase increases NIX levels, promoting mitosis (42). The interaction between mitochondrial-bound NIX and autophagosome-localized LC3 forms a mitochondria-NIX-LC3-autophagosome complex, which has been implicated in excessive mitophagy following spinal cord injury (SCI). Inhibiting NIX may serve as a neuroprotective strategy in such cases (43). In NIX^-/-^ platelets, MMP decreases, mitochondrial reactive oxygen species (mtROS) levels rise, oxygen consumption declines, and adenosine triphosphate (ATP) production is impaired, leading to platelet dysfunction and increased risk of thrombosis (44). In patients with ulcerative colitis, the absence of NIX impairs the clearance of damaged or dysfunctional mitochondria in the intestinal epithelium, exacerbating the disease through dysregulated mtROS production (45).

FUNDC1 is a complete OMM protein with an N-terminal LC3-interacting region (LIR) motif, serving as a receptor for hypoxia-induced mitophagy (46). Under normal physiological conditions, FUNDC1-mediated mitophagy is regulated by phosphorylation at Tyr18 by SRC kinase and at Ser13 by casein kinase 2 (CK2), as well as by the inhibition of phosphatase activity through the interaction of Bcl2-like 1 (BCL2L1) with phosphoglycerate mutase 5 (PGAM5). Hypoxia stimulation leads to the degradation of BCL2L1, activating PGAM5, which catalyzes the dephosphorylation of FUNDC1 at Ser13, thereby increasing the interaction between FUNDC1 and LC3 (47). Mutations or deletions in the LIR motif of FUNDC1 impair its ability to mediate mitophagy. In cells lacking ATG5, FUNDC1-mediated mitophagy, particularly in response to hypoxia, is significantly inhibited, indicating that this process is dependent on ATG5 (48). Under hypoxic conditions, FUNDC1-mediated upregulation of mitophagy activates the ROS-HIF1α pathway, promoting the proliferation of pulmonary artery smooth muscle cells, which ultimately leads to pulmonary vascular remodeling and hypoxic pulmonary arterial hypertension (49). The mitochondrial E3 ligase MARCH5 degrades FUNDC1, reducing mitochondrial sensitivity to hypoxia-induced mitophagy (50). Dysfunction in mitochondrial quality control caused by FUNDC1 deficiency in adipose tissue exacerbates diet-induced obesity and metabolic syndrome through MAPK activation, white adipose tissue remodeling, and subsequent inflammatory responses (51). Exercise increases FUNDC1 expression, which helps prevent coronary endothelial cell aging and protects elderly mice from myocardial ischemia/reperfusion injury. Additionally, FUNDC1 plays a protective role in neurons following SCI by inducing mitophagy, inhibiting mitochondrial-dependent apoptosis, and enhancing mitochondrial function (52).

Prohibitin 2 (PHB2) and PHB1 together form the mitochondrial inhibitory protein complex, where PHB2 directly binds to LC3-II, and PHB1 interacts with LC3-II through PHB2. A significant downregulation of PHB2 leads to a reduced rate of mitochondrial clearance (53). In patients with cholestatic liver injury, hepatocellular mitophagy induced by bile acids requires the interaction of PHB2 with LC3 and SQSTM1 (54). An increase in MicroRNA-24-3p levels can downregulate PHB2, which helps inhibit autophagy in myocardial fibroblasts and alleviates myocardial fibrosis (55). Under high glucose conditions, TIPE1 destabilizes PHB2 in renal tubular epithelial cells, promoting mitophagy and exacerbating tubular injury (56). Conversely, overexpression of PHB2 promotes mitophagy and delays aging in mouse myocardial cells (57).

In addition, the AMP-activated protein kinase (AMPK)/mammalian target of rapamycin (mTOR) s pathway plays a critical role in shifting cells from synthetic metabolism to catabolic metabolism and serves as a key regulatory pathway for autophagy. ULK1, a conserved substrate of AMPK, is involved in this process. Activation of AMPK promotes mitophagy by enhancing mitochondrial division (58).

## Lipid based mitophagy

Mitochondrial lipids, such as cardiolipin (CL), ceramide, and sphingosine-1-phosphate (S1P), serve as mitophagic signals that facilitate the clearance of damaged mitochondria by interacting with the mitophagic machinery (59).

In healthy mitochondria, CL plays a crucial role in lipid–protein interactions necessary for mitochondrial function (60). LC3 possesses a putative CL binding site at its N-terminal α-helices, which is crucial for the direct interaction between the OMM and the mitophagosome, ultimately facilitating mitophagy (61). Ceramide selectively targets mitochondria to LC3B-II-containing autophagolysosomes. LC3B-phosphatidylethanolamine is lipidated to form LC3B-II, which then binds to ceramide on the OMM, indicating that the ceramide–LC3B-II interaction is a key factor in triggering lethal mitophagy (62). S1P is directly involved in LC3 lipidation and interacts with PHB2, playing a significant role in the regulation of mitophagy (63).

### Micromitophagy

Micromitophagy is a mechanism for the targeted removal of damaged mitochondrial components through the formation of mitochondria-derived vesicles (MDVs) that bud off and are subsequently transported to lysosomes (64). MDVs are cargo-selective vesicles released from mitochondria independently of the mitochondrial fission machinery. Oxidative stress stimulates MDVs formation, and these vesicles are enriched with oxidized mitochondrial proteins (65). Although MDVs formation and transit to lysosomes occur independently of the autophagic proteins ATG5 and LC3, this process requires the involvement of PINK1 and Parkin (66). Ultimately, MDVs fuse with lysosomes to complete the hydrolysis and degradation of their contents.

### Transmitophagy

Transmitophagy is a specialized mode of mitochondrial degradation in neurons with long axons. Under normal physiological conditions, axonal mitochondria of long-projection neurons are enclosed in axoplasmic membranes, which are then shed and degraded by neighboring cells (67). This process, where neuronal mitochondria are internalized and degraded by astrocytes, is known as transmitophagy (68). Mitochondria can traverse cell boundaries (69), and in neurons with long axons, they may be transferred to adjacent astrocytes for degradation (67). A significantly greater proportion of retinal ganglion cell mitochondria are degraded in the optic nerve head (ONH) than in the cell soma. Some axonal mitochondria are not degraded cell-autonomously within retinal ganglion cell axons via traditional mitophagy but are instead processed by resident astrocytes through a process termed transcellular degradation of mitochondria, or transmitophagy (70). The internalization of neuronal mitochondria is notably increased in astrocytes isolated from Alzheimer’s disease (AD) mouse brains, indicating the presence of neuron-astrocyte transmitophagy in AD (68). It has also been shown that the mitophagy of degenerating dopaminergic terminals begins in dopaminergic spheroids and concludes in the surrounding astrocytes. Neuron-astrocyte transmitophagy is critical for preventing the release of damaged mitochondria into the extracellular space and for mitigating the neuroinflammatory activity characteristic of Parkinson’s disease (PD) (71).

### Mitophagy in neurons

The human brain, despite accounting for only 2% of the body’s volume, is responsible for 25% of its oxygen consumption. These high energy demands make the brain particularly vulnerable to damage during acute or chronic hypoxia or ischemia. Bioenergy depletion is also a known contributor to neuronal death in a range of neurodegenerative diseases. Beyond ATP production, neuronal mitochondria play a crucial role in Ca^2+^ buffering (3). The high demand for mitochondria in neurons is evident from the elevated mitochondrial density at presynaptic endings, postsynaptic densities, nodes of Ranvier, and growth cones, all of which rely on mitochondrial function to sustain neuronal activity (72). As neurons are non-proliferating cells that do not undergo mitosis, their mitochondria are especially susceptible to accumulating oxidative damage over time. Additionally, high levels of Ca^2+^ influx into neurons may exacerbate this stress. Unsurprisingly, mitochondrial damage and mitophagy dysfunction are linked to several age-related neurodegenerative diseases, including PD, AD, and Huntington’s disease (HD) (73).

## Role of mitophagy in programmed neuronal death

**Figure 2:**
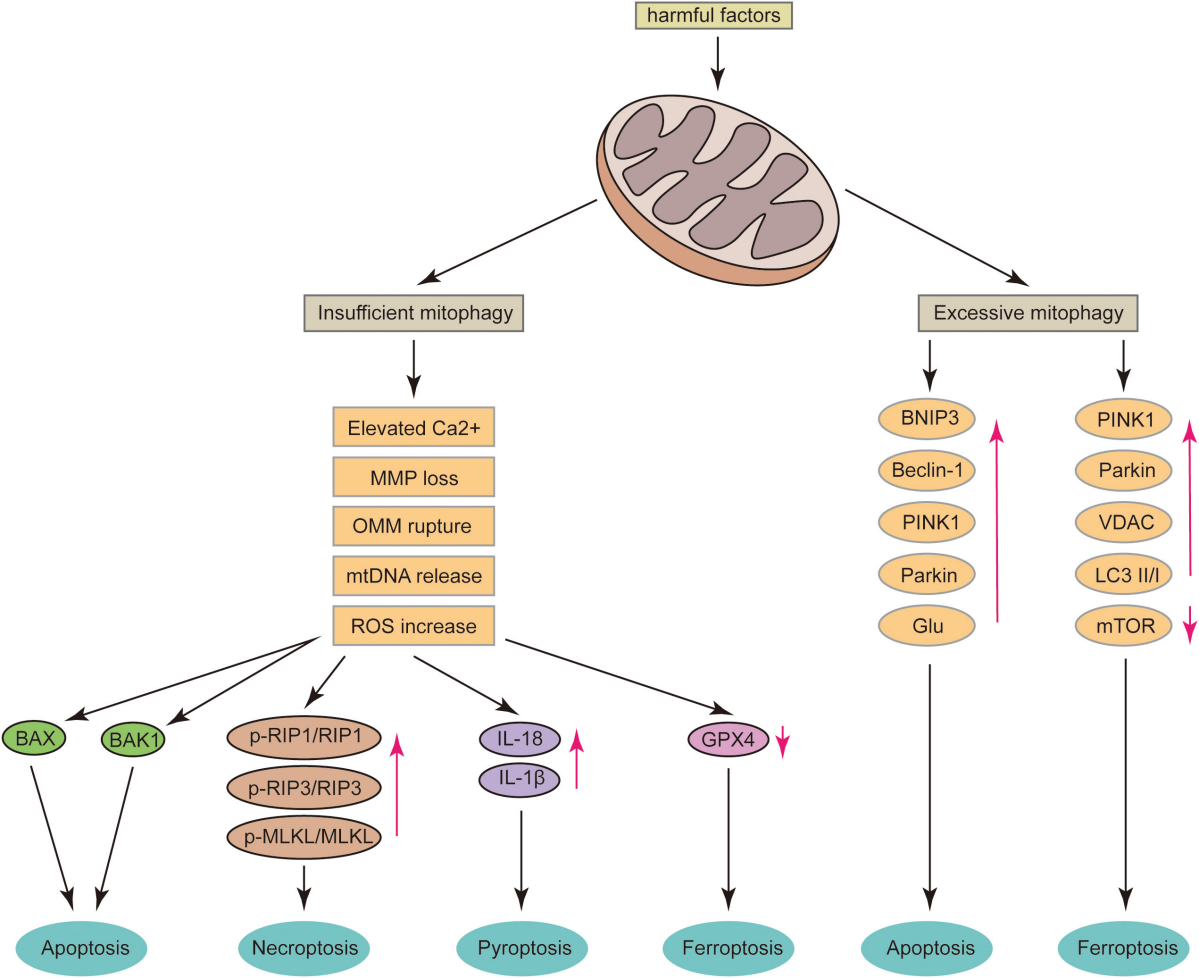
After mitochondrial damage, several critical events can occur, including the release of mtDNA, rupture of the OMM, loss of MMP, and an increase in Ca^2+^ levels. If mitophagy is insufficient, these conditions can lead to neuronal apoptosis via the BAX and BAK1 pathways; increase the ratios of p-RIP1/RIP1, p-RIP3/RIP3, and p-MLKL/MLKL, thereby promoting neuronal necroptosis; elevate neuronal IL-18 and IL-1β levels, inducing pyroptosis; and significantly reduce GPX4 levels, leading to ferroptosis. Conversely, excessive mitophagy can result in the overexpression of BNIP3, Beclin-1, PINK1, Parkin, and Glu, which may induce neuronal apoptosis. It can also cause the overexpression of PINK1, Parkin, and VDAC, increase the LC3 II/I ratio, decrease mTOR levels, and ultimately induce neuronal ferroptosis.

While limited neuronal death is a highly regulated and necessary homeostatic mechanism for maintaining the functional development of the central nervous system, pathological neuronal loss in mature central nervous systems can lead to irreversible declines in motor and cognitive functions (74). Programmed neuronal death, including apoptosis, necroptosis, pyroptosis, and ferroptosis, is closely associated with central nervous system (CNS) diseases, including acute CNS injuries such as intracerebral hemorrhage, subarachnoid hemorrhage, traumatic brain injury, and neurodegenerative diseases like AD and PD. Mitochondria play a pivotal role in CNS diseases by mediating various cell death pathways (75). The regulation of mitophagy is critical in controlling ROS levels, mtDNA release, OMM rupture, MMP loss, and Ca^2+^ imbalance in neurons (**Figure 2**). Moderate mitophagy is essential for maintaining mitochondrial function and cellular homeostasis. Therefore, understanding the relationship between mitophagy and programmed neuronal death is crucial.

### Mitophagy and neuronal apoptosis

#### Mechanisms of mitophagy and neuronal apoptosis

Apoptosis, also known as programmed cell death, is a self-destructive cellular mechanism involving multiple biological events. Mitochondria play a crucial role in regulating apoptosis pathways. Apoptosis depends on the Bcl-2 protein family, with two key pro-apoptotic proteins, Bcl-2 associated X protein (BAX) and Bcl-2 antagonist killer 1 (BAK1), which induce cell apoptosis through OMM (76). BAX and BAK1 are essential for neuronal apoptosis (77, 78), and they interact with VDAC2 to ensure their ability to penetrate the OMM and exert their effects (79, 80). In healthy cells, cytochrome c functions as a component of the electron transport chain within the mitochondrial intermembrane space. Upon mitochondrial damage, the permeability of the OMM increases, leading to the release of apoptotic factors such as cytochrome c. The depletion of cytochrome c in mitochondria increases ROS production, exacerbating mitochondrial dysfunction and promoting cell apoptosis. Mitophagy plays a protective role by transporting defective mitochondria to lysosomes for degradation, eliminating ROS, and inhibiting apoptosis (81, 82). Excessive accumulation of Ca^2+^ in the mitochondrial matrix can cause matrix expansion, leading to the rupture of the OMM and the subsequent release of cytochrome c (83). Additionally, the release of mtDNA can act as a trigger for inflammation. PINK1/Parkin-mediated mitophagy prevents the release of mtDNA into the cytoplasm by removing damaged mitochondria, thereby alleviating cGAS/STING-induced neuroinflammation and neurodegeneration (84).

#### Targeted treatment to mitophagy-mediated neuronal apoptosis

Activating PINK1/Parkin-mediated mitophagy can mitigate neuronal apoptosis induced by prion PrP106-126 (20) and cadmium (85). Compounds such as salidroside (Sal) (86), DHA (87), and triiodothyronine (T3) (88) have been shown to enhance the PINK1/Parkin pathway. Additionally, DJ-1 can activate the ERK1/2 pathway and improve mTOR signaling in dopaminergic neurons (89); Tissue-type plasminogen activator (tPA) increases AMPK phosphorylation and boosts FUNDC1 expression (90), while the LncRNA MEG3 promotes FUNDC1 expression through the Rac1-ROS axis (91). Furthermore, dexmedetomidine enhances the PINK1/Parkin pathway by activating AMPK (92). These pathways collectively promote mitophagy, reduce ROS production, prevent mitochondrial-dependent neuronal apoptosis, and exert neuroprotective effects. 2,2’,4,4’-Tetrabromodiphenyl ether (PBDE-47) induces mitochondrial abnormalities by impairing PINK1/Parkin-mediated mitophagy, leading to excessive apoptosis and, consequently, promoting neuronal loss and subsequent neurobehavioral defects. Melatonin counteracts PBDE-47-induced damage by activating the AMPK/ULK1 signaling pathway, thereby restoring mitophagy and preventing neuronal apoptosis (93).

### Mitophagy and neuronal necroptosis

#### Mechanisms of mitophagy and neuronal necroptosis

Necroptosis is a form of regulated programmed cell necrosis. Tumor necrosis factor-α (TNF-α) is the most widely studied inducer of necroptosis. Upon binding to its receptor, TNF-α induces a conformational change in the TNF receptor, which recruits several proteins to the cytoplasmic portion of the receptor. These proteins include Tumor necrosis factor receptor type 1-associated death domain protein (TRADD), receptor-interacting serine/threonine-protein kinase 1 (RIPK1), cellular inhibitor of apoptosis-1 (cIAP1), cIAP2, and TNFα receptor-associated factors (TRAFs). In the absence of caspase 8, these proteins form membrane complexes that induce the formation of RIPK1-RIPK3 necrosomes. This leads to mitochondrial hyperpolarization, lysosomal membrane permeabilization, and ROS production, ultimately activating Mixed Lineage Kinase Domain-Like protein (MLKL), which disrupts the plasma membrane and results in cell death (94). Necroptosis is closely associated with ATP depletion, ROS accumulation, calcium overload, and the opening of mitochondrial permeability transition pores (MPTP) (95). For example, increased expression of RIPK3 can suppress the AMPK pathway and inhibit Parkin-mediated mitophagy. The loss of mitophagy enhances the opening of mitochondrial permeability transition pores (MPTP), ultimately leading to cellular necroptosis (96). Tricalcium phosphate (TCP) has been shown to enhance the kinase activity of RIP1, RIP3, and MLKL, thereby promoting cell necroptosis. ROS scavengers can inhibit the increase in the p-RIP1/RIP1, p-RIP3/RIP3, and p-MLKL/MLKL ratios caused by TCP particles (97). Mitochondria, as the primary organelles responsible for ATP production and a major source of ROS, play a crucial role in necroptosis. Mitophagy, regulated by PINK1, influences the expression of MLKL and, consequently, necroptosis (98).

#### Targeted treatment to mitophagy-mediated neuronal necroptosis

RIPK3 can reduce Parkin phosphorylation levels, thereby decreasing the interaction between Parkin and LC3, which is typically induced by hypoxic injury. This inhibition of mitophagy by RIPK3 plays a critical role in cell death mechanisms. During ischemia-reperfusion (I/R) injury, RIPK1 is phosphorylated, leading to the subsequent phosphorylation of RIPK3, which promotes the activation of MLKL. Once activated, MLKL executes the cell death program. PGAM5, a mitochondrial membrane protein, acts as an important protective gene against ischemic injury. It serves as an anchor for the RIP1-RIP3-MLKL complex within mitochondria, promoting mitophagy and protecting cells from necroptosis (99). Sterile alpha and toll/interleukin 1 receptor motif-containing protein 1 (SARM1) is a central determinant of axonal degeneration. Its upregulation enhances the RIPK1-RIPK3-MLKL signaling axis, thereby promoting neuronal necroptosis. Rapamycin has been shown to enhance mitophagy and alleviate neuronal necroptosis, particularly in conditions involving SARM1 aggregation, such as acrylamide-induced dying-back neuropathy (100). In patients with PD, ROS generation and mitochondrial depolarization can trigger neuronal necroptosis. This process can be inhibited by inactivating RIP1. Necrostatin-1 (Nec-1), a known inhibitor of RIP1 kinase activity, increases the levels of TOMM20 and PHB1 proteins, which are involved in mitochondrial function. This increase is accompanied by the upregulation of LONP1, a mitochondrial protease that plays a role in the PINK1-dependent mitophagy pathway. By enhancing mitophagy, Nec-1 prevents MLKL phosphorylation and the subsequent plasma membrane rupture associated with p-MLKL, thereby mitigating neuronal necroptosis (101).

### Mitophagy and neuronal pyroptosis

#### Mechanisms of mitophagy and neuronal pyroptosis

Pyroptosis is a recently identified pro-inflammatory mode of cell death, triggered by various inflammation-related caspases. The assembly and activation of inflammasome complexes in response to intracellular and extracellular pathological signals lead to the activation of inflammatory caspases 1, 4, 5, and 11, ultimately causing mitochondrial outer membrane permeability (MOMP) and subsequent cell death (102). The hallmark event of pyroptosis involves the processing of IL-18 and IL-1β, and the activation of the pore-forming protein gasdermin-D (GSDMD), which culminates in cell membrane rupture and the release of IL-18 and IL-1β (103). While canonical GSDMD is cleaved by caspases 1, 4, 5, and 11, another variant, gasdermin-E (GSDME), is cleaved by caspase-3, linking non-inflammatory apoptosis to pyroptosis. GSDME-dependent pyroptosis also results in the release of pro-inflammatory cytokines IL-1β and IL-18 (104). Current research suggests a negative feedback loop between mitophagy and pyroptosis (105). Inflammasome-mediated activation of caspase-1 inhibits mitophagy, aggravating mitochondrial damage. Conversely, the absence of the key mitophagy regulator Parkin exacerbates mitochondrial damage, promoting pyroptosis (106). This mechanism may be associated with pyroptosis-induced mitochondrial ROS release and disruption of membrane integrity. Additionally, potassium efflux and cytochrome c are crucial in the regulation of mitophagy and pyroptosis (107).

#### Targeted treatment to mitophagy-mediated neuronal pyroptosis

Anesthesia and surgery can lead to insufficient PINK1-mediated mitophagy, which in turn activates the caspase-3/GSDME pyroptosis signaling axis, ultimately resulting in postoperative cognitive dysfunction (POCD). Overexpression of PINK1 has been shown to alleviate cognitive impairment and reduce caspase-3/GSDME-dependent pyroptosis (108). SNAP25 plays a key role in promoting PINK1-dependent mitophagy, rescuing defects in the PINK1/Parkin pathway, facilitating the conversion of LC3-I to LC3-II, preventing abnormal accumulation of P62, and suppressing the activation of the caspase-3/GSDME axis. These actions collectively hinder neuronal pyroptosis and offer neuroprotection against POCD (109). Tumor necrosis factor α-induced protein 1 (TNFAIP1) impairs mitophagy and triggers excessive neuronal pyroptosis by inhibiting SNAP25 expression. A deficiency in TNFAIP1 function enhances PINK1/Parkin-dependent mitophagy in HT22 cells, thereby preventing caspase-3/GSDME-dependent pyroptosis. Conversely, increased TNFAIP1 function inhibits mitophagy and promotes pyroptosis (110). PINK1/Parkin-mediated mitophagy may act as an endogenous neuroprotective mechanism during the pathological progression of cerebral ischemia/reperfusion (CI/R) by modulating NLRP3-mediated pyroptosis. Glycosides have been shown to activate key mitophagy markers, such as LC3-II/LC3-I and P62, and the PINK1/Parkin mitophagy pathway, which alleviates neuronal pyroptosis following middle cerebral artery occlusion/reperfusion (MCAO/R) induced oxygen-glucose depletion/reoxidation (OGD/R) in rats (111). Following traumatic brain injury (TBI), levels of IL-1β, IL-18, cleaved caspase-1, GSDMD, and TNF-α increase. The expression of the PINK1/Parkin pathway is enhanced by human umbilical cord mesenchymal stem cell-derived exosomes, significantly inhibiting the levels of pro-inflammatory cytokines and improving secondary neuronal pyroptosis after TBI (112). Dexmedetomidine has been shown to reduce postoperative cognitive impairment in aged rats by promoting PINK1-mediated mitophagy and suppressing caspase-1/GSDMD-induced pyroptosis in hippocampal neurons (113).

### Mitophagy and neuronal ferroptosis

#### Mechanisms of mitophagy and neuronal ferroptosis

Ferroptosis is an iron-dependent form of cell death characterized by the toxic accumulation of lipid peroxides on the cell membrane (114). This process is marked by the loss of activity of the lipid repair enzyme glutathione peroxidase 4 (GPX4), accompanied by the buildup of lipid ROS (115). The production of mitochondrial ROS can promote lipid peroxidation, leading to ferroptosis; however, treatment with mitochondria-targeted lipophilic antioxidants can significantly rescue cells from GPX4 inactivation-induced ferroptosis (116). ATP consumption activates AMPK, which effectively inhibits ferroptosis by phosphorylating and inactivating acetyl-CoA carboxylase (ACC) (117). Additionally, mitochondrial biosynthetic products involved in cellular metabolism influence the ferroptosis pathway (118). Mitophagy reduces ferroptosis through the ROS/heme oxygenase-1 (HO-1)/GPX4 axis. Inhibition of BNIP3/Parkin or PINK1/Parkin-mediated mitophagy exacerbates ROS release, lipid peroxidation, and cellular ferroptosis (119). Moreover, the phosphorylation of FUNDC1 can disrupt mitophagy and mitochondrial quality control, ultimately leading to cellular ferroptosis (120). Dihydroorotate dehydrogenase (DHODH), a mitochondrial enzyme located on the outer surface of the mitochondrial inner membrane, plays a crucial role in this process. Coenzyme Q (CoQ) is primarily synthesized in mitochondria, and DHODH is well-positioned to reduce CoQ to CoQH2, thereby exerting its anti-ferroptosis function within mitochondria. When GPX4 is inactivated, DHODH can detoxify lipid peroxides and prevent mitochondrial ferroptosis (118).

#### Targeted treatment to mitophagy-mediated neuronal ferroptosis

Following TBI, the expression of PINK1 and Parkin significantly increases. Exosome treatment further enhances the expression of PINK1 and Parkin in neurons, while decreasing ACSL4 expression and increasing GPX4 levels in the treatment group (112). This suggests that human umbilical cord mesenchymal stem cell-derived exosomes reduce neuronal ferroptosis through PINK1/Parkin-mediated mitophagy, providing neuroprotection in TBI (112). Caveolin-1 (Cav-1) regulates the mitochondrial fission-mitophagy axis to maintain mitochondrial quality, thereby alleviating neuronal ferroptosis and significantly improving diabetes-associated cognitive dysfunction (DACD) (121). Acteoside (ACT) activates the Nrf2-mitophagy axis, upregulates GPX4 and XCT, reduces lipid peroxidation, and mitigates ferroptosis. ACT treatment has been shown to preserve dopaminergic neurons, curb ferroptosis in these cells, and alleviate cognitive and behavioral deficits in PD (122). DR-Ab, an antibody targeting the DR-region of the Na+/K+-ATPase (NKA)α subunit, disrupts the cytosolic interaction between NKAα1 and Parkin, facilitating Parkin’s translocation to mitochondria and enhancing mitophagy. DR-Ab also promotes the formation of the surface NKAα1/XCT complex, thereby inhibiting XCT-dependent ferroptosis. Given NKAα1’s role as a key regulator of ferroptosis and mitophagy, its DR-region presents a promising therapeutic target for PD (123).

### Excessive mitophagy induces neuronal death

In neurological diseases, most secondary neuronal apoptosis is attributed to insufficient mitophagy, while in some cases, the pathogenesis is linked to excessive mitophagy (**Table 1**). Excessive mitophagy can exacerbate mitochondrial damage, leading to subsequent neuronal apoptosis and ferroptosis (124). NIX primarily regulates basal levels of mitophagy under physiological conditions, whereas BNIP3 exclusively triggers excessive mitophagy, resulting in cell death (125). Inhibiting BNIP3 may protect hippocampal neural cells from OGD/R, reduce excessive mitophagy caused by CI/R injury, maintain mitochondrial integrity, reduce neuronal apoptosis, and improve neurological function (126). Sirtuin 1 (SIRT1), a NAD+-dependent deacetylase, induces mitophagy by deacetylating downstream targets such as Mfn2. Inhibiting SIRT1 expression can alleviate mitophagy and suppress neuronal apoptosis following CI/R (127). FBXL4 mutations lead to excessive mitophagy through the accumulation of BNIP3/BNIP3L, resulting in mitochondrial DNA depletion and dysfunction in cortical neurons (128). Chronic cerebral hypoperfusion (CCH) significantly decreases the expression of proteins such as P62, CTSD, and LAMP1, while increasing the expression of beclin-1, Parkin, and BNIP3, as well as the LC3-II/LC3-I ratio, and the release of cytochrome c from mitochondria to the cytoplasm. These changes induce lysosomal dysfunction, promote autophagic volume accumulation, and lead to excessive autophagy in neurons, ultimately triggering neuronal apoptosis (129). Melatonin can reduce the levels of mitophagy proteins PINK1 and Parkin, decrease the colocalization of Tom20 and LC3, alleviate neuronal hypoxia after stroke, and improve post-stroke cognitive impairment (PSCI) (130). The basolateral amygdala (BLA), a key structure in encoding emotional valence and responding to stress and threats, can experience excessive elimination of mitochondria due to overexpression of PINK1 and Parkin, resulting in anxiety in stressed mice (131). Excess glutamate (Glu) can induce excessive mitophagy by activating the glutamate receptor 2 (GluR2)-Parkin pathway, leading to mitochondrial dysfunction, apoptosis of hippocampal neurons, a deficiency in monoamine neurotransmitters, and the development of diabetes-related depression (DD) in rats (132). Tris (1,3-dichloro-2-propyl) phosphate (TDCPP) enhances iron ion influx into mitochondria and mitochondrial depolarization via activation of VDAC channels, triggering excessive mitophagy through the PINK1/Parkin pathway, leading to mitophagy-related ferroptosis and TDCPP-induced neurotoxicity (133). Exposure to fine particulate matter (PM2.5) significantly upregulates HO-1, which mediates PM2.5-induced mitophagy-dependent ferroptosis in hippocampal neurons. The upregulation of HO-1 increases the LC3-II/I ratio, decreases mTOR expression, and leads to excessive autophagy, exacerbating ferroptosis in PM2.5-exposed hippocampal neurons. Inhibiting mitophagy or ferroptosis may be key therapeutic targets to mitigate neurotoxicity following PM2.5 exposure (134).

**Table 1:** Diseases caused by excessive mitophagy leading to neuronal death.

Disease	Modle	Death Type	Mechanisms	Refs
CI/R	HT22 mouse cells	Apoptosis	BNIP3 overexpression	(126)
Cortical neuron dysfunction	Male mice	Apoptosis	FBXL4 mutations	(128)
CCH	Male rats	Apoptosis	Overexpression of Beclin-1, Parkin, and BNIP3	(129)
PSCI	Male mice	Apoptosis	Elevated levels of PINK1 and Parkin	(130)
Anxiety	stressed mice	Apoptosis	excessive increase of PINK1 and Parkin in BLA	(131)
DD	Male rats	Apoptosis	Overactivated GluR2-Parkin pathway	(132)
TDCPP-induced neurotoxicity	Male mice and HT22 mouse cells	Ferroptosis	Overexpression of PINK1/Parkin pathway, VDAC channels activation	(133)
PM2.5-induced neurotoxicity	Male mice and HT22 mouse cells	Ferroptosis	Increased LC3 II/I ratio and decreased mTOR expression	(134)

### Mitophagy in neurodegeneration

Neurodegenerative diseases (NDDs) are a spectrum of complex, heterogeneous disorders characterized by the progressive degeneration of neurons, affecting both the central and peripheral nervous systems (135). Two regions of the brain particularly vulnerable to oxidative damage are the hippocampus and the substantia nigra (SN). Hippocampal degeneration is a hallmark of diabetic encephalopathy and AD, while SN degeneration is characteristic of PD (136). Selective neurodegeneration in AD and PD is associated with increased oxidative stress markers in these brain regions (137). At the organellar level, mitochondrial dysfunction is a prominent phenotype in neurodegenerative diseases. Additionally, the accumulation of damaged mitochondria in most NDDs suggests dysregulation of the mitophagy pathway (135). Biologically, AD is defined by two principal neuropathological hallmarks: the abnormal accumulation of extracellular amyloid-β (Aβ) plaques and intracellular tau-containing neurofibrillary tangles (138). Insufficient mitochondrial function and bioenergy output in AD patients may lead to reduced cellular energy levels, while the concomitant leakage of electrons promotes the formation of ROS. These ROS can damage proteins, membrane lipids, and nucleic acids. By initiating membrane lipid peroxidation, mitochondrial ROS may also promote the accumulation of pathological extracellular Aβ peptides and intraneuronal hyperphosphorylated tau protein, leading to the formation of AD-defining Aβ plaques and neurofibrillary tangles, which further exacerbate mitochondrial defects (139, 140). Mitophagy reduces AD-related tau hyperphosphorylation and prevents cognitive impairment, indicating that impaired clearance of defective mitochondria is a key event in AD pathogenesis and that mitophagy could be a potential therapeutic target (141). Impairment of mitophagy was initially associated with PD based on observations that brain samples from PD patients showed accumulation of autophagosomes containing damaged mitochondria (142). Additionally, mutations in PINK1 and Parkin, which are critical proteins involved in mitophagy, have been linked to the early onset of autosomal recessive PD (143). Neuronal mitochondrial dysfunction and disruptions in the mitophagy pathway are significant contributors to the onset of PD. Huntington’s disease (HD) is a neurodegenerative disorder caused by mutations in the gene encoding the huntingtin protein. The mutant huntingtin protein does not affect the PINK1/Parkin pathway but disrupts the interaction of mitophagy adaptors OPTN, CALCOCO2, SQSTM1/P62, and NBR1 with LC3, leading to impaired fusion of mitochondria and autophagosomes and a compromised mitophagy mechanism (144). This damage is partially recovered by overexpressing PINK1, which improves mitochondrial integrity and protects neural function (145).

## Role of mitophagy in neuroinflammation

Neuroinflammation refers to the inflammatory response in the CNS triggered by brain or spinal cord injury, infection, ischemia, diabetes, intraocular pressure, or as a consequence of autoimmunity and aging. This inflammation is primarily driven by the release of proinflammatory cytokines, chemokines, second messengers such as nitric oxide and prostaglandins, and ROS (146). The core basis of neuroinflammation are likely consistent across various conditions, including aging, metabolic diseases like hypertension and diabetes, and cerebral insults such as stroke and injury. The inflammatory mediator TNF-α has been identified as a key player and biomarker of neuroinflammation (147). Inflammatory signals are typically responses to pathogens or foreign substances. However, new evidence suggests that mitochondria or mitochondrial components—including mtDNA, mitochondrial transcription factor A (TFAM), cardiolipin, cytochrome c, formyl peptides, high mobility group B protein 1 (HMGB1), and ATP—can mimic pathogens and trigger injury responses (148). Therefore, the role of mitophagy in neuroinflammation warrants further discussion.

### Microglial mitophagy in neuroinflammation

Microglia are ubiquitously distributed in the brain and serve as the principal innate immune cells, acting as the first responders to pathological insults (149). These cells express a variety of receptors, including the CD200 receptor, colony-stimulating factor 1 receptor, chemokine receptor (CX3CL1), neurotrophic factors, and neurotransmitters, all of which play significant roles in neuroinflammation (150). Upon activation by endogenous stimuli generated after injury or infection, inhibitor of NF-κB (i-κB), which is bound to nuclear factor-kappa B (NF-κB) in the cytoplasm of microglia, is phosphorylated and degraded by i-κB kinase. This process causes NF-κB to translocate to the nucleus, where it promotes the transcription of pro-inflammatory cytokine genes (151). Damaged neurons also release fractalkine (CX3CL1) ligands, which are recognized by the CX3CL1 receptor on microglia (152). Activated microglia can exhibit different gene expression patterns, with the M1 phenotype associated with pro-inflammatory effects and the M2 phenotype with anti-inflammatory effects (153). The surface of microglia contains Aβ receptors, such as NOD-like receptors (NLRs), Toll-like receptors (TLRs), and receptors for advanced glycation end products (RAGE). Aβ can penetrate the cell membrane of microglia, bind to the intracellular domain of NLRs, and activate inflammasomes containing NOD-, LRR-, and pyrin domain-containing 3 (NLRP3). Aβ can also bind to RAGE, leading to the release of pro-inflammatory cytokines such as IL-1β and TNF-α (154). Mitochondria play a critical role in neuroinflammation. When microglia are treated with mitochondrial lysates, triggering receptor expressed on myeloid cells 2 (TREM2) expression decreases, while TNF-α expression increases, along with elevated levels of MMP-8 and IL-8, redistribution of NF-κB towards the nucleus, and increased phosphorylation of p38 MAPK. mtDNA directly obtained from mitochondria has been shown to increase TNF-α mRNA levels in microglia. These findings suggest that at least one mitochondrial-derived damage-associated molecular pattern (DAMP) molecule, mtDNA, can induce inflammatory changes in microglia and neuronal cell lines, thereby triggering neuroinflammation (148). The ability of mitophagy induction to reduce neuroinflammation may be explained by several mechanisms and benefits in microglia. First, the accumulation of damaged mitochondria releases DAMPs, including increased ROS production, decreased ATP levels, and elevated oxidative stress (155). Second, blockage of mitophagy has been shown to increase ROS levels, which in turn activates the NLRP3 inflammasome (156). Additionally, the mitophagy inducer mitochonic acid 5 (MA-5) has been reported to reduce neuroinflammation. Treatment of microglia with MA-5 leads to improved mitochondrial quality, dependent on the mitophagy activator Bcl2/BNIP3 (157). Collectively, mitophagy may inhibit neuroinflammation through multiple pathways.

Recent studies have increasingly shown that mitophagy in microglia can alleviate neuroinflammation, which is believed to be a contributing factor to AD. Inadequate mitophagy is partly implicated in the pathogenesis of AD. As a receptor involved in mitophagy, OPTN can mitigate neuroinflammation through the AIM2 and RIPK1 pathways, and a deficiency in OPTN may be a potential factor leading to the development of AD (154). Treatment with T3 enhances mitophagy through the PINK1/Parkin pathway, facilitating the degradation of damaged mitochondria, reducing ROS release, and subsequently decreasing microglial cell aggregation and activation. This process helps alleviate neuroinflammation following subarachnoid hemorrhage (SAH) (88). Copper exposure has been shown to activate microglia, leading to the secretion of inflammatory products, early activation of the ROS/NF-κB pathway, and subsequent mitophagy disorders in microglial cells, ultimately resulting in microglia-mediated neuroinflammation (158). Mitophagy disorders are characterized by excessive mtROS production, which has been observed in overactivated primary microglia and BV-2 cells, driving microglial polarization toward the M1 pro-inflammatory state. Mitophagy deficiency in microglia not only exacerbates neuroinflammation but also impairs their phagocytic function, leading to further neuronal damage (159). Compounds such as Nrf2 (160), divanillyl sulfone (DS) (161), quercetin (Qu) (162), and polygala saponins (PSS) (163) have been shown to promote mitophagy in microglia, improve the accumulation of ROS from dysfunctional mitochondria, inhibit the activation of the NLRP3 inflammasome, and alleviate neuroinflammation. In a sleep apnea mouse model, NLRP3 deficiency was found to protect against intermittent hypoxia-induced neuroinflammation by promoting the PINK1/Parkin mitophagy pathway in microglia (164). Additionally, inhibiting the activation of the HMGB1/RAGE axis increases stress-induced mitophagy flux, thereby reducing microglia-mediated neuroinflammation (165).

### Astrocyte mitophagy in neuroinflammation

Although initially considered to have only passive functions, recent studies have revealed that astrocytes play active and essential roles in maintaining brain homeostasis (166). Pro-inflammatory reactive astrocytes upregulate several genes, such as those involved in the complement cascade, and induce the production of pro-inflammatory factors like IL-1β, TNF-α, and NO (167). Inflammatory mediators secreted by microglia, including IL-1α, IL-1β, TNF-α, and C1q, can activate pro-inflammatory astrocytes (168). When primary astrocytes and microglia are activated by cytokines, there is an upregulation of intracellular Ca^2+^ mobilization and nuclear factor of activated T cells (NFAT) activation. NFAT is positively regulated by IL-1β, and in a positive feedback loop, IL-1β expression is dependent on NFAT and L-type Ca^2+^ channels (169). Mitophagy helps reduce cellular Ca^2+^ and NFAT levels by clearing damaged mitochondria (3, 170). Additionally, the activation of astrocytes can mediate a neuroinflammatory response in dopaminergic neurons through the generation of ROS and lipid peroxidation (171). Mitophagy can alleviate mitochondrial injury caused by ROS accumulation and lipid peroxidation (172).

There are currently limited studies on the role of astrocyte mitophagy in neuroinflammation. Manganese treatment significantly reduces Mfn2 mRNA levels in astrocytes and induces neuroinflammation by impairing mitochondrial dynamics, further supporting the role of mitophagy dysfunction in mediating astrocyte-induced neuroinflammation (173). ATP-sensitive potassium (K-ATP) channels, which couple cell metabolism to membrane potential, are crucial in this context. Kir6.1, a pore-forming subunit of the K-ATP channel, is prominently expressed in astrocytes and plays a role in regulating their function. The astrocytic Kir6.1/K-ATP channel has been shown to promote mitophagy. In the substantia nigra compacta of Kir6.1 knockout mice, the expression of p65, activation of caspase-1, and levels of IL-1β, NLRP3, and TNF-α in response to MPP+ stimulation were significantly higher in astrocytes from Kir6.1 KO mice compared to those from wild-type mice (174). Resolvin D1 has been found to enhance mitophagy by activating ALX4/FPR2 receptors on astrocytes, thereby protecting mitochondria in primary astrocytes and ultimately guarding against neuroinflammation after TBI (175). Benzopyrene (BaP), associated with cognitive decline and an increased risk of neurodegenerative diseases, may contribute to these conditions by inhibiting PINK1/Parkin-mediated mitophagy, leading to neuroinflammation in the brain (176).

### Neuronal mitophagy in neuroinflammation

Neuronal cell death is a significant driver of neuroinflammation. Traditionally, the relationship between neurons and inflammation is viewed with inflammation acting upon neurons. However, neurons can also actively contribute to inflammation, creating a bi-directional relationship where neurons are both recipients and instigators of inflammation (177). Evidence suggests that neuronal apoptosis can disrupt the homeostatic microglial phenotype, further influencing inflammatory responses (178). Other forms of cell death, such as necroptosis and pyroptosis, have more direct consequences for neuroinflammation. Lytic cell death, as seen in necroptosis and pyroptosis, leads to the release of intracellular components that act as DAMPs, which incite further inflammation (179). Neurons undergoing pyroptotic cell death also release potent inflammatory cytokines such as IL-1β (180). Similarly, activation of necroptosis has been linked to the activation of the NLRP3 inflammasome and the release of IL-1β (181). When neuronal cells are treated with mitochondrial lysate, levels of TNF-α and NF-κB protein increase, while I-κB protein levels decrease, indicating a heightened inflammatory response (148). Treatment with the mitophagy inducer urolithin A has been shown to improve synaptic connectivity and reduce neuroinflammation in neurons, highlighting the importance of mitophagy in resolving cytosolic mtDNA-triggered inflammation (182). Considering that mitophagy can inhibit neuronal necroptosis and pyroptosis, mitophagy plays a protective role against neuron-induced neuroinflammation.

## Conclusion and future perspective

In this article, we explored the relationship between mitophagy and both programmed neuronal death and neuroinflammation. We also examined the potential interplay between mitophagy, programmed neuronal death, and neuroinflammation. Additionally, we briefly discussed the role of mitophagy in neurodegeneration. Furthermore, we highlighted potential targets for future clinical treatments aimed at addressing neuronal death and neuroinflammation.

Programmed neuronal death and neuroinflammation play crucial roles in both acute central nervous system injuries and chronic degenerative diseases. Numerous studies have shown that mitochondrial damage and dysfunction are closely linked to these processes. Mitophagy regulates the quality and quantity of mitochondria in neurons, making its dysfunction or overactivation significantly related to neuronal death and neuroinflammation. In most cases, the activation of mitophagy has a protective effect on damaged neurons and supports cell survival. However, in instances where cell death is mediated by excessive mitophagy, therapeutic interventions that further enhance mitophagy may not rescue the phenotype. Therefore, it is essential to study the relationship between mitochondrial dysfunction and disease progression across various disease states and to explore how mitochondrial homeostasis can be achieved through mitophagy, thereby potentially delaying or treating neurological diseases. Moreover, there is currently no literature indicating whether excessive mitophagy leads to neuronal necroptosis and pyroptosis, warranting further research in this area. Additionally, no experiments have yet investigated whether neuronal apoptosis and ferroptosis contribute to neuroinflammation, nor has the relationship between mitophagy, programmed neuronal death, and neuroinflammation been fully established. Further research is needed to explore these critical aspects.

Although studies have shown that autophagy benefits neurons by reducing damage and death, and have explored its potential mechanisms at the cellular level, no research has yet determined the optimal extent to which autophagy should be controlled to achieve the best outcomes. Further investigation in this area is necessary. Specifically, more research at the cellular level is needed to understand the mechanisms and physiology of mitophagy in mitigating programmed neuronal death and neuroinflammation. While numerous animal and cell experiments have demonstrated the potential therapeutic role of mitophagy in neurological disease models, there are currently no relevant agonists that have been applied to humans. It remains unclear whether these agents would have similar effects in human subjects. Additionally, the pharmacology and genetics of mitophagy as a treatment for neurological diseases are still being explored in animal and cellular studies, with no clinical evidence available yet. Furthermore, no animal research has determined whether enhancing neuronal function could potentially harm other organs or systems. Therefore, future experiments should investigate whether treating neurological diseases with mitophagy agonists might lead to secondary damage to other organs or systems. Continued efforts are required to translate these findings into clinical research and to elucidate the potential mechanisms involved.
